# Progress in Obstetrics

**Published:** 1901-08-24

**Authors:** 


### Progress in Obstetrics.

**Post Partum Hæmorrhage.**—E. Stanmore Bishop,[1] writing on this subject, strongly advocates compression of the abdominal aorta in the treatment of post partum hæmorrhage. He says that in ninety-nine cases out of a hundred the hæmorrhage is due to exhaustion of the uterine muscle. Before any tired muscle can fulfil its function again properly it must have rest and a reasonable amount of blood circulating in it, and this end can be attained by compression of the abdominal aorta. The objection that is sometimes raised that compression of this vessel does not completely control the blood supply of the uterus is, he maintains, rather an advantage than otherwise, as by compressing the aorta you control the uterine arteries which convey five-sixths of the blood to the uterus, while the ovarian vessels which convey the remaining sixth will keep up a sufficient supply of blood to preserve the vitality of the organ, assist in the formation of small plugs in the open arterial mouths, and at the same time vivify and strengthen the muscular tone, which temporarily is at too low an ebb, so that when once the full force of the blood current is allowed to bear upon them the vessels shall be found closed by clot and constricted by the now firmly contracting fibres around. As to the length of time during which the compression should be kept up, this will depend upon the rapidity with which the uterus regains its tone. When the organ, as ascertained by gently touching it from time to time, is found to be tonically contracted, the compressing hand is very slowly and gradually lifted, and the contraction now established, being a natural one, will be found to be permanent.

**Primary Ovarian Pregnancy.**—G. P. Anning and H. Littlewood [2] report an interesting and instructive case of primary ovarian pregnancy in which rupture occurred 14 days after the last menstruation. The patient was operated upon about thirty-six hours after the rupture. About two pints of blood and clot were removed, and a small ovum about the size of a Barcelona nut was found. There was a rent in the right ovary leading to a cavity which contained some blood; and into this the ovum and its sac exactly fitted, clearly indicating the primary ovarian origin of the pregnancy. The right tube was removed and showed no evidence of rupture. The left tube was examined and was found to be normal. Bland-Sutton [3] discussing this case remarked that the condition of parts was exactly analogous to that of a tubal mole, and might appropriately be called an "ovarian mole." The blood corpuscles in the vessels of the villi were nucleated; there was no trace of a decidua in the ovarian follicle; and it was an interesting fact that in the clinical report of the case it was stated that a decidua had formed in the uterus, and a few days after the operation it was discharged. A study of this case also opens up a large field of inquiry as to the origin of the so-called blood cysts of the ovary.

**Puerperal Eclampsia.**—Dr. Jardine [4] writing on this subject says that with increasing experience he is more confident than ever that saline diuretic infusion is the best method of treating this formidable disease, his aim being to dilute the poison and get rid of it as quickly as possible. He reports six cases of marked dropsy and albuminuria of pregnancy with threatening convulsions, in which by prompt purging with salts, the use of diuretics, and a milk diet, all except one escaped eclampsia. If, he argues, by the establishment of diuresis one can prevent the fits, surely it is reasonable to suppose they can be cured by the same method. When the fits are occurring diuresis cannot be established quickly enough in the ordinary way; absorption by the stomach is almost in abeyance, hence the great advantage of giving the drugs subcutaneously. Besides the diuretic effect, we get a dilution of the poison and a stimulation of the patient. As to the obstetric treatment of the condition Dr. Jardine advises that if labour has not begun, we should not interfere. During the first stage, if the fits cease dilatation may be left to nature, but if they recur the uterus should be emptied as quickly as possible. During the second stage delivery should be effected at once. W. Stroganoff [5] considers puerperal eclampsia to be an acute infective disease which usually runs its course in a few hours, rarely exceeding twenty-four, and still more rarely forty-eight hours. In most cases the convulsions constitute the greatest danger from their effect upon the heart, the respiratory centre, and the kidneys. If the convulsions can be stopped, or their intensity diminished, the resisting power of the patient's organism will usually suffice to nullify the effect of the offending germs. Therefore, the prevention or amelioration of the convulsions is the main object of treatment. Dr. G. Elder [6] says that it is now generally recognised that eclampsia is due to some form of auto-intoxication, the vast majority of cases following upon renal insufficiency either as an expression of a passing congestion or organic disease; and he maintains that if it became the routine practice for the urine of pregnant women to be examined at short intervals from the fourth month to the end of pregnancy, and treatment adopted at the first indication of kidney failure, many valuable lives would be saved. Dr. Dewar,[7] discussing the etiology of eclampsia, says he believes the convulsions are due to a poison, the product of a bacillus, and that albuminuria may or may not be present. As regards treatment, he condemns the use of morphia, and advocates an expectant treatment with the administration of bromides and chloral. If at the onset of convulsions labour had not commenced, he would not interfere, but when uterine contractions are present and the os beginning to dilate, he would empty the uterus as soon as possible.

**Tumours Complicating Pregnancy and Labour.**—Pregnancy and labour complicated with the presence of a tumour often places the life of both the mother and child in extreme danger, especially if the pregnancy be advanced to full term, and the tumour situated in the lower segment of the uterus. With regard to the treatment of ovarian tumours complicating labour, Bland Sutton [8] in a very instructive paper on this subject, condemns craniotomy, forceps, and version, and says that the choice of treatment really lies between two methods: (*a*) In the early stage to push the tumour out of the pelvis and allow labour to be completed, and then at some convenient season to perform ovariotomy; (*b*) to perform ovariotomy at once and then to accelerate labour by the use of forceps. If the tumour cannot be removed, then perform Cæsarian section. Of these two methods of treatment he favours the latter, and considers that reposition of the tumour exposes the mother to two very grave risks, viz., rupture of the cyst, and acute twisting of the pedicle. When pregnancy and a fibroid tumour of the uterus co-exist, and a careful examination discloses the fact that if the pregnancy goes to term, delivery by the natural passages would be impossible, he considers abdominal hysterectomy to be the best treatment. When labour is actually in progress and an impacted fibroid is found obstructing delivery, he recommends that Cæsarian section should be performed, and the uterus and tumour afterwards removed. With regard to that most appalling of all the complications of pregnancy, viz., cancer of the cervix, he recommends that when the pregnany has gone to term, and the child is living, Cæsarian section should be performed. When, however, the cancerous condition of the cervix is detected before the mid-period of gestation, it would be desirable to empty the uterus and peform vaginal hysterectomy, provided the disease is not too extensive. On the other hand, when the disease is not discovered until a later period the cervix may be removed without disturbing the pregnancy. Gillaume and Keiffer [9] report an interesting case where a fibroid mass reached to the epigastrium and blocked the pelvis, the patient having advanced to the fifth month of pregnancy. Keiffer removed the whole mass above the cervix and the patient made an uninterrupted recovery. John Duncan Emmet [10] records a case in which he removed nine myomata during pregnancy. The patient bore the operation well, and the pregnancy continued to full term when she was delivered of a well-developed healthy girl. A case of Cæsarian section is recorded by G. L. Brodhead.[11] The operation was performed on account of the presence of a fibro-cystic tumour of the uterus which obstructed delivery; the tumour which was attached to the posterior wall of the uterus was afterwards removed, and the site of its attachment to the uterus closed with fine catgut sutures. Recovery was rapid and uneventful.

[1] Lancet, April 13, 1901. [2] Lancet, Jan. 12, 1901. [3] Lancet, Jan. 12, 1901. [4] Brit. Med. Jour., March 2, 1901. [5] Amer. Gynæcol. and Obstet. Jour., April, 1901. [6] Brit. Med. Jour., March 2, 1901. [7] Edin. Med. Jour., January, 1901. [8] Lancet, Feb. 9, 16, and 23, 1901. [9] Brit. Med. Jour., Jan. 19, 1901. [10] Amer. Gynæcol. and Obstet. Jour., June, 1901. [11] The Post Grad., March, 1901.

